# Etude de facteurs de risque de la transmission du VIH de la mère à l'enfant dans la stratégie « option A » à Lubumbashi, République Démocratique du Congo

**DOI:** 10.11604/pamj.2015.22.18.7480

**Published:** 2015-09-09

**Authors:** Dieudonné Tshikwej Ngwej, Olivier Mukuku, Rachel Mudekereza, Eugénie Karaj, Etienne Bwana Fwamba Odimba, Oscar Numbi Luboya, Jean-Baptiste Sakatolo Kakoma, Stanis Okitotshio Wembonyama

**Affiliations:** 1 Département de Pédiatrie, Faculté de Médecine, Université de Lubumbashi, RD Congo; 2 Bureau provincial de ICAP/RDC, Lubumbashi, RD Congo; 3 Département de Chirurgie, Faculté de Médecine, Université de Lubumbashi, RD Congo; 4 Département de Gynécologie-Obstétrique, Faculté de Médecine, Université de Lubumbashi, RD Congo

**Keywords:** VIH, TME, transmission verticale, facteurs de risque, Lubumbashi, HIV, PMTCT, vertical transmission, risk factors, Lubumbashi

## Abstract

**Introduction:**

L'infection à VIH chez la femme enceinte a pour principal risque la contamination du nouveau-né. L'objectif est de déterminer le taux de transmission du VIH de la mère à l'enfant (TME) dans la ville de Lubumbashi et en évaluer les facteurs de risque.

**Méthodes:**

Il s'agissait d'une étude prospective transversale à visée analytique de 157 accouchées séropositives au VIH et de leurs enfants dans 12 structures sanitaires de Lubumbashi (RDCongo) du 1er octobre 2012 au 31 décembre 2013. Les paramètres sociodémographiques, cliniques et les données relatives aux activités de PTME du VIH ont été étudiés. Les statistiques usuelles ont été utilisées pour analyser les résultats. Le seuil de significativité a été fixé à une valeur de p < 0,05.

**Résultats:**

Le taux de transmission verticale du VIH était de 12,7% (20/157). Il n'y avait pas d'association significative entre les caractéristiques sociodémographiques maternelles telles que l’âge, la parité, le niveau d’étude, la profession et l’état-civil et la TME (p>0,05). La transmission verticale du VIH était significativement associée aux facteurs suivants: le stade clinique 3 de l'OMS (OR=5,18 (1,5-18,1)), la présence d'infection opportuniste (OR=8,7 (2,7-27,8)), le dépistage lors de l'accouchement (OR=6,3 (1,0-39,0)) ou au cours de l'allaitement (OR=7,1 (1,1-76,7)), au taux de CD4 maternel <350/mm^3^ (OR=2,9 (1,1-7,7)), l'absence de thérapie antirétrovirale chez la mère (OR=19,9 (4,8-81,9)), la naissance avant terme (OR=4,7 (1,4-16,0)), la rupture prématurée de membranes (OR=45,0 (7,4-454,6)), le faible poids de naissance (OR=5,6 (1,9-16,7)), la notion de réanimation néonatale (OR=12,4 (3,8-40,1)), la non administration de la névirapine à la naissance (OR=26,4 (7,6-92,3)) et l'alimentation mixte (OR=12,6 (1,3-115,9)). Le sexe du nourrisson et le mode d'accouchement n’étaient pas non plus associés à la transmission verticale du VIH (p>0,05).

**Conclusion:**

Le taux de TME du VIH demeure fort élevé à Lubumbashi comme dans la plupart de pays en développement où les nouveau-nés continuent d’être infectés par le VIH de manière verticale alors que certains facteurs de transmission sont tout à fait souvent évitables. La réduction de cette transmission passe par une amélioration du système de suivi des grossesses dans notre milieu.

## Introduction

Depuis plusieurs années, la lutte contre le virus de l'immuno-déficience humaine (VIH) est un enjeu de santé publique. La transmission verticale du VIH est la principale voie par laquelle les enfants sont infectés par le VIH. Une femme infectée par le VIH peut transmettre le virus à son bébé pendant la grossesse, le travail, l´accouchement ou l´allaitement. En l´absence de toute intervention, le risque combiné de la transmission de la mère à l'enfant (TME) du VIH in utero et pendant l´accouchement est de 15-30% et le risque est accru chez les enfants allaités à 20-45% [1–3]. Cette transmission verticale est très élevée dans les pays à ressources limitées, ce qui fait de la prévention de la transmission du VIH de la mère à l'enfant (PTME) une intervention prioritaire des programmes de lutte contre le VIH/SIDA et demeure un challenge dans la majorité de ces pays, particulièrement en Afrique [4]. Globalement, l'Organisation Mondiale de la Santé (OMS) estime que 3,3 millions d´enfants vivaient avec le VIH en 2012 [5], étaient infectés principalement par voie verticale et 90% de ces infections avait eu lieu en Afrique subsaharienne [2]. Dans les pays développés, le risque de TME du VIH a été réduit à moins de 1%, grâce à la large diffusion de l´administration du traitement antirétroviral (TARV) chez les femmes enceintes et chez les enfants exposés au VIH et au recours systématique à l'allaitement artificiel [5].

Au niveau mondial, plus de 966 000 femmes enceintes dans les pays à faible revenu et à revenu intermédiaire ont reçu le traitement antirétroviral (en trithérapie ou en prophylaxie) en 2013, soit 67% de l'ensemble des femmes enceintes VIH+ dans ces pays [6]. En conséquence, l'OMS souligne que moins d´enfants contractent le VIH: dans les 21 pays prioritaires d´Afrique du Plan mondial, le taux global de transmission de la mère à l´enfant a diminué, passant d´environ 26% en 2009 à 17% en 2012, et plus de 800 000 infections à VIH chez les enfants ont été empêchées pendant ces quatre années [6]. En République Démocratique du Congo (RDC), la prévalence nationale du VIH chez les femmes enceintes varie entre 3,7 et 4,3% [7, 8] et à Lubumbashi, chez les femmes enceintes du site sentinelle de la séro-surveillance, elle est de 4,6% (IC95%: 2,9-7,1%) [7]. En 2011, la ville de Lubumbashi comptait 25 sites de PTME sur 251 structures sanitaires où les femmes accouchent, soit une couverture de 9,9% [9, 10]; mais aucune donnée n'est disponible, à l'exclusion de la ville de Lubumbashi, quant à l'impact de la stratégie PTME dans la réduction de la TME.

Plusieurs études récentes ont identifié les facteurs de risque de la transmission verticale du VIH qu'il faut observer dans la mise en œuvre d'un programme de PTME. Ces études ont réparti en 5 catégories ces facteurs: (1) les facteurs maternels (par exemple l’état immunologique de la mère, le traitement anti-rétroviral), (2) les facteurs virologiques (par exemple la charge virale), (3) facteurs obstétricaux (par exemple l'accouchement traumatique, la rupture prématurée des membranes, la chorioamniotite), (4) les facteurs fœtaux (par exemple la prématurité) et (5) les facteurs du nourrisson (par exemple l´état immunitaire, TARV, l´allaitement, la nutrition) [11]. C'est ainsi que nous avons mené cette étude dont les objectifs sont de déterminer le taux de TME et d'identifier les facteurs de risque de TME du VIH, dans un programme de PTME « option A », à Lubumbashi (République Démocratique du Congo (RDC)).

## Méthodes

### Type d’étude et cadre d'intervention

Il s'agit d'une étude prospective transversale à visée analytique menée sur la période allant du 1er octobre 2012 au 31 décembre 2013. L’étude a été réalisée dans 12 structures sanitaires (CS) réparties dans 6 zones de santé (ZS) de la ville de Lubumbashi en RDC (ZS de Lubumbashi, ZS de Kamalondo, ZS de Mubunda 1, ZS de Mubunda 2, ZS de Katuba et ZS de Kisanga). Ces 12 structures de santé sont les suivantes: CS Sainte Bernadette, CS Saint Charles, CS Saint François, CS Betty, CS Chrina Médical, CS Baraka, CS Amani, CS Mama wa Umoja, CS Mubunda 1, CS Mubunda 2, Cliniques Universitaires et Hôpital Général de référence de Kisanga. Elles ont été choisies car elles font partie des institutions appuyées par l'organisation non gouvernementale (ONG) internationale « ICAP ». Cette ONG fournit à ces structures un appui logistique conséquent notamment en intrants (tests de dépistage, TARV, suivi biologique, outil de rapportage de données); de même, tous les agents de santé de ces structures ont été formés dans la PTME selon le protocole du programme national en vigueur et dans la récolte et la gestion de données ayant servi au présent travail.

### Population d’étude

Cette étude multicentrique a ciblé toutes les femmes enceintes ou allaitantes séropositives vues, enregistrées et suivies par les services de PTME dans les structures sanitaires ci-haut citées. Ces gestantes et mères ont été dépistées VIH positifs pendant les consultations prénatales (CPN), au moment de l'accouchement ou lors de consultations préscolaires (CPS). Après le dépistage, chaque gestante ou mère séropositive était appelée à poursuivre les CPN, à accoucher et à suivre les CPS dans les structures où elle avait été dépistée. Mais celle qui n'avait pas suivi le circuit prévu, était récupérée soit lors de l'accouchement soit lors des CPS. Toutes les gestantes et mères séropositives inscrites dans le programme de PTME de ces structures ont bénéficié d'un suivi gratuit de la part des agents de santé en collaboration avec ceux de l'ONG ICAP. Ce suivi concerne les consultations, les examens paracliniques (comptage du nombre de lymphocytes CD4, dépistage précoce de l'enfant au VIH, …) ainsi que la thérapie antirétrovirale pour le couple mère-enfant.

### Schémas ARV

Le régime antirétroviral utilisé au cours de la période d´étude a été celui adopté à l´échelle nationale et dicté par le protocole national de PTME qui correspond à l’ « option A » de l'OMS [12, 13]. Cette option A consiste en l'administration de la trithérapie ARV (zidovudine (AZT) + lamivudine (3TC) + névirapine (NVP)) chez les femmes éligibles (c'est-à-dire celles qui sont aux stades cliniques 3 ou 4 de l'OMS ou celles dont le nombre de CD4 ≤350 cellules/mm3); et chez les femmes non éligibles (c'est-à-dire celles qui sont aux stades cliniques 1 ou 2 de l'OMS ou celles qui ont un nombre de CD4 >350 cellules/mm^3^), une prophylaxie antirétrovirale (ARV) était administrée en monothérapie, bithérapie ou trithérapie en fonction du moment de dépistage. Cette prophylaxie ARV consiste en: AZT (300 mg) tous les jours pendant la période prénatale à partir de la 14^ème^ semaine; AZT + 3TC (600/300 mg en une prise) et NVP (dose unique 200 mg) au cours du travail et de l'accouchement; AZT + 3TC (300/150 mg) deux fois par jour pendant 7 jours après l'accouchement. Pour les nourrissons allaités au sein, la prophylaxie ARV donnée à la mère doit être associée à l'administration quotidienne au nourrisson de NVP suspension (2mg/kg) à partir de la naissance et poursuivie jusqu’à une semaine après l'arrêt de toute exposition au lait maternel. Pour les nourrissons qui ne sont pas allaités au sein, la prophylaxie ARV donnée à la mère doit être associée à l'administration quotidienne au nourrisson de NVP suspension (2mg/kg) à partir de la naissance jusqu’à l’âge de 6 semaines (moment de dépistage).

### Dépistage du VIH chez le nourrisson

Conformément aux directives nationales, les nourrissons ont passé leur premier test de dépistage par la méthode de la réaction en chaîne par polymérase de l´ADN (Polymerase Chain Reaction (PCR-ADN)) par Spot sang séché à l’âge de 6 semaines ou à l'opportunité la plus proche pour ceux qui ont été vus après 6 semaines. La transmission de l'infection à VIH a été définie comme résultat positif à la première PCR-ADN réalisée et recontrôlée [14]. La séronégativité au VIH était confirmée au premier test PCR-ADN chez les enfants non allaités au lait maternel ou au test réalisé après arrêt de tout allaitement maternel. Ainsi, l’âge des nourrissons à la confirmation du statut sérologique à VIH variait de 1,5 mois à 16 mois. Au total, 157 couples mère-enfant chez qui le statut sérologique final des enfants a été confirmé ont été suivis et enregistrés au cours de la période d’étude.

### Collecte des données et paramètres étudiés

Les données en rapport avec la mère et son nourrisson ont été recueillies sur une fiche individuelle préétablie. Du point de vue maternel, les informations recueillies ont été: l’âge, la parité, le stade clinique selon l'OMS, la présence d'infections opportunistes, le nombre de lymphocytes CD4, le moment de dépistage, la période de mise sous TARV et le TARV reçu (classé en mono-, bi- et trithérapie). Le terme de la grossesse (semaines d'aménorrhée (SA)), le moment de la rupture des membranes (en dehors du travail ou pendant le travail), le mode d'accouchement (voie basse ou césarienne) ont été également notés. Pour l'enfant, les informations recueillies ont été: l’âge de l'enfant lors de la confirmation du statut sérologique, le sexe, le poids de naissance, la notion de réanimation à la naissance, le type d'allaitement avant le dépistage, l'administration de la névirapine en monothérapie à la naissance et le résultat sérologique VIH.

### Analyses statistiques

L'analyse des données a été réalisée à l'aide des logiciels Epi-Info 2011 et Stata 12. Le statut sérologique à VIH de l'enfant est considéré ici comme variable dépendante et les paramètres en rapport avec les caractéristiques maternelles, anténatales et néonatales constituent les variables indépendantes. Les variables indépendantes des enfants dépistés séropositifs au VIH ont été comparées à celles des enfants dépistés séronégatifs, à l´aide du test du khi2 corrigé de Yates ou du test exact de Fisher (lorsque recommandé) pour les variables qualitatives (comparaison de proportions) dans une série d´analyses univariées et à l´aide du test de Student pour les variables quantitatives (comparaison de moyennes). Les facteurs atteignant un degré de signification de p < 0,05 ont été considérés comme facteurs de risque de la transmission verticale présentés avec leurs odds ratio (OR) et intervalles de confiance à 95% (IC à 95%).

### Considérations éthiques

La recherche pour réaliser ce travail a été autorisée par le comité d’éthique de l'Université de Lubumbashi et par l'autorité sanitaire de la province du Katanga. Un consentement libre et éclairé (verbal ou écrit) de toutes les personnes impliquées dans cette étude a été obtenu au préalable.

## Résultats

### Taux de TME

Des 157 enfants enregistrés dans notre étude, 20 ont été infectés par le VIH soit un taux de TME de 12,7%.

### Caractéristiques maternelles

***Paramètres sociodémographiques***: le Tableau 1 montre la répartition des caractéristiques maternelles sociodémographiques en fonction de l’état sérologique des enfants. Ni l’âge maternel, ni la parité, ni l’état-civil, ni la profession, ni le niveau d’étude n'ont montré une quelconque influence sur la TME (p>0,05).

**Tableau 1:** Caractéristiques maternelles sociodémographiques

Variable	Enfants VIH+ (n=20)		Enfants VIH- (n=137)		Total (n=157)		p	OR [IC95%]
	n	%	n	%	n	%		
**Age maternel**								
<20 ans	2	25	6	75	8	100	0,2567	2,5 [0,2-15,7]
20-39 ans	16	11,6	122	88,4	138	100	-	1
≥40 ans	2	18,2	9	81,8	11	100	0,6236	1,7 [0,2-9,3]
Moyenne	29,5±7,3		30,0±6,1		29,9± 6,3		0,7321	
**Parité**								
Primipare	5	23,8	16	76,2	21	100	0,1993	2,5 [0,8-7,8]
Multipare	15	11,0	121	89,0	136	100	-	1
Moyenne	3,6±2,4		3,8±2,1		3,8±2,1		0,7121	
**Etat-civil**								
Mariées	18	14,1	110	85,9	128	100	0,3726	2,2 [0,5-10,1]
Singletons	2	6,9	27	93,1	29	100	-	1
**Niveau d’étude**								
Analphabète/ Primaire	13	16,9	64	83,1	77	100	0,1975	2,1 [0,8-5,6]
Secondaire/ Universitaire	7	8,8	73	91,3	80	100	-	1
**Profession**								
Sans occupation	16	15,0	91	85,0	107	100	0,5499	1,6 [0,5-4,6]
Occupées	5	10,0	45	90,0	50	100		1

***Paramètres clinique, paraclinique et thérapeutique***: le Tableau 2 montre la distribution des paramètres maternels clinique, paraclinique et thérapeutique en fonction de l’état sérologique des enfants. En ce qui concerne la stadification clinique de l'OMS, plus l'on passe d'un stade inférieur à un stade supérieur, plus le taux de TME augmente: 6,7%, 17,4% et 27,3% respectivement aux stades cliniques 1, 2 et 3. Aucune différence statistique significative n'est mise en évidence entre les deux premiers stades (p=0,1039). Mais entre le premier et le troisième, l'analyse statistique montre une différence significative (p=0,0166) signifiant un risque multiplié par 5,18 pour qu'un enfant de notre série né d'une mère séropositive au stade 3 pût être infecté (OR=5,18; IC95%: 1,48-18,13).

**Tableau 2:** Paramètres maternels clinique, para clinique et thérapeutique

Variable	Enfants VIH+ (n=20)		Enfants VIH- (n=137)		Total (n=157)		p	OR [IC95%]
	n	%	n	%	n	%		
**Stade clinique de l'OMS**								
1	6	6,7	83	93,3	89	100		1
2	8	17,4	38	82,6	46	100	0,1039	2,9 [0,9-9,0]
3	6	27,3	16	72,7	22	100	**0,0166**	5,2 [1,5-18,1]
**Infection opportuniste**								
Absente	13	9,2	129	90,8	142	100		1
Présente	7	46,7	8	53,3	15	100	**<0,0001**	8,7 [2,7-27,8]
**Moment de dépistage**								
Avant la grossesse	2	5,0	38	95,0	40	100		1
Au cours de la grossesse	8	9,4	77	90,6	85	100	0,4997	2,0 [0,4-19,9]
A l'accouchement	4	25,0	12	75,0	16	100	**0,0493**	6,3 [1,0-39,0]
Pendant l'allaitement	6	37,5	10	62,5	16	100	**0,0193**	7,1 [1,1-76,7]
**Taux de CD4**								
<350/mm^3^	11	21,6	40	78,4	51	100	**0,0407**	3,0 [1,1-7,7]
≥350/mm^3^	9	8,5	97	91,5	106	100		1
Moyenne	377,5±228,5		469,7±249,2		457,9±247,9		0,1207	
**Période de mise sous TARV**								
Avant la grossesse	1	3,1	31	96,9	32	100		1
Au cours de la grossesse	4	4,8	80	95,2	84	100	1,000	1,6 [0,2-14,4]
Lors de l'accouchement ou après	4	23,5	13	76,4	17	100	**0,0431**	9,5 [1,0-93,7]
Non mise sous TARV	11	45,8	13	54,2	24	100	**<0,0001**	26,2 [3,1-224,5]
**Type de TARV reçu**								
Monothérapie	3	4,4	65	95,6	68	100		1
Bithérapie	1	12,5	7	87,5	8	100	0,3652	3,1 [0,3-33,9]
Trithérapie	4	7,0	53	93,0	57	100	0,7011	1,6 [0,3-11,6]
Aucun	12	50,0	12	50,0	24	100	**<0,0001**	21,9 [4,7-130,7]

TARV: traitement antirétroviral

En ce qui concerne les infections opportunistes dans la TME, 46,9% des mères qui en avaient au cours de la grossesse ou lors de l'accouchement ont transmis le VIH chez leurs enfants versus 9,2% chez celles qui n'en avaient pas; l'analyse statistique montre une différence significative entre ces deux proportions. Le risque pour qu'une mère séropositive de notre série présentant une infection opportuniste au cours de la grossesse ou lors de l'accouchement transmette le VIH chez son enfant était de 8,68 fois (OR=8,68; IC95%: 2,71-27,80). Des 40 et 85 mères dépistées respectivement avant et au cours de la grossesse, les proportions respectives de TME étaient de 5% et 9,4% alors que ces fréquences étaient respectivement de 25% et 37,5% chez les mères dépistées à l'accouchement et pendant l'allaitement. En comparant la période d'avant et pendant la grossesse à celle d'accouchement et après en ce qui concerne le dépistage, la différence est statistiquement significative (p < 0,05). En ce qui concerne la période de mise sous TARV, la période per ou post-partale et la non mise sous ARV s’étaient avérées plus à risque pour la TME comparativement à la période antépartale. Les mères mises sous TARV avant la grossesse ou pendant la grossesse avaient transmis le VIH à leurs enfants moins que celles ayant reçu le TARV après ou lors de l'accouchement ou celles qui n'en ont pas reçu (3,1% et 4,8% versus 23,5% et 45,8%; p < 0,05).

La moyenne du taux de CD4 maternel était de 377,5±228,5/mm^3^ (extrêmes: 54 et 1060/mm^3^) dans le groupe d'enfants séropositifs alors qu'elle était de 469,7±249,2/mm^3^ (extrêmes: 41 et 1320/mm^3^) dans le groupe d'enfants séronégatifs. Bien que la différence entre ces deux moyennes ne soit pas statistiquement significative (t=1,56; p=0,1207), la comparaison des proportions de <350/mm^3^ et de <350/mm^3^ montre une différence statistique significative (21,6% versus 8,5%; p=0,0407) signifiant un risque de TME de 2,96 fois élevé pour une mère dont le taux de CD4 est <350/mm^3^ (OR=2,96; IC95%: 1,14-7,70). Quant au type de TARV reçu, aucune différence statistique n'est mise en évidence entre la mono-, la bi- et la trithérapie (p < 0,05); mais quant à la comparaison de la proportion des mères ayant reçu la monothérapie (4,4%) avec celle des mères ne l'ayant pas reçu (47,8%), elle montre que ces proportions sont très significativement différentes par rapport à la TME (OR=19,86; IC95%: 4,81-81,95; p < 0,001).

### Caractéristiques partiales

La distribution des caractéristiques partales en fonction de l’état sérologique des enfants est présentée dans le Tableau 3. L’âge gestationnel moyen était de 37,6 ± 2,6 SA (extrêmes: 30 et 40 SA) dans le groupe d'enfants séropositifs alors qu'il était de 38,6 ± 1,7 SA (extrêmes: 28 à 42 SA) pour les séronégatifs; la comparaison de ces deux moyennes donne une différence statistiquement significative (t=2,27; p=0,0246). Il en est de même pour la comparaison entre la proportion des enfants nés à terme et celle des enfants nés avant terme (10,5% versus 35,7%; p=0,0225). Les enfants nés avant terme présentent 4,7 fois plus de risque d’être infecté que ceux nés à terme (OR=4,7; IC95%:1,4-16,0). S'agissant de la rupture de membranes, la TME était de 80% chez les mères dont les membranes se sont rompues prématurément alors que cette fréquence était de 8,2% chez les mères dont la rupture des membranes est survenue au cours du travail (OR=45,0; IC95%: 7,4-454,6; p < 0,001). Concernant la voie d'accouchement, aucune différence statistique significative n'est mise en évidence (p=0,4234).

**Tableau 3:** Caractéristiques partiales

Variable	Enfants VIH+ (n=20)		Enfants VIH- (n=137)		Total (n=157)		P	OR [IC95%]
	n	%	n	%	n	%		
**Age gestationnel**								
<37 SA	5	35,7	9	64,3	14	100	0,0225	4,7 [1,4-16,0]
≥37 SA	15	10,5	128	89,5	143	100		1
Moyenne	37,6±2,6		38,6±1,7		38,5±1,9		0,0246	
**Rupture des membranes**								
Prématurée	8	80,0	2	20,0	10	100	<0,0001	45,0 [7,4-454,6]
Au cours du travail	12	8,2	135	91,8	147	100		1
**Mode d'accouchement**								
Voie basse	19	12,4	134	87,6	153	100		1
Césarienne	1	25,0	3	75,0	4	100	0,4234	2,4 [0,1-30,8]

### Caractéristiques des enfants

Le Tableau 4 montre la répartition des caractéristiques des enfants en fonction de leur état sérologique. L’âge moyen des enfants au moment du diagnostic était de 4,3±3,6 mois (extrêmes: 1,5 et 16 mois); il était de 4,1±3,1 mois chez les nourrissons non infectés contre 5,8±3,9 mois chez les nourrissons infectés par le VIH (p=0,0528). S'agissant du sexe, aucune différence statistique n'est mise en évidence (p=0,1130). La moyenne du poids de naissance était de 2746,5±566,2 grammes (extrêmes: 1770 et 3750 grammes) dans le groupe d'enfants séropositifs alors qu'elle était de 3027,9±505,9 grammes (extrêmes: 1700 et 5400 grammes) dans le groupe d'enfants séronégatifs. L'analyse statistique donne une différence significative entre ces deux moyennes (t=2,28; p=0,0235). La proportion de faible poids de naissance chez les enfants infectés est significativement plus élevée que celle de normopondérés infectés (36,8% versus 9,4%; p=0,0027). Les enfants nés avec un poids de moins de 2500 grammes présentent un risque de TME de 5,6 fois supérieur par rapport à ceux nés avec un poids ≥2500 grammes (OR=5,6; IC95%:1,9-16,7). Cinquante-trois pourcents des enfants ayant été réanimés à la naissance ont été infectés par le VIH contre 8,5% de ceux qui ne l'ont pas été; la comparaison de ces deux fréquences donne une différence très hautement significative (p < 0,001) signifiant un risque d'infection multiplié par 12,4 fois pour un enfant issu d'une mère infectée par le VIH et réanimé à la naissance (OR=12,4; IC95%: 3,8-40,1). Les enfants qui n'avaient pas reçu de la névirapine à la naissance (10/15) ont été plus infectés que ceux qui en avaient reçu (10/142) et présentent un risque de 26,4 fois d’être infecté (OR=26,4; IC95%: 7,6-92,3; p < 0,001).

**Tableau 4:** Caractéristiques des enfants

Variable	Enfants VIH+ (n=20)		Enfants VIH- (n=137)		Total (n=157)		p	OR [IC95%]
	n	%	n	%	n	%		
**Age**								
Moyenne (mois)	5,8±3,9		4,1±3,1		4,3±3,6		0,0528	
Extrêmes (mois)	1,5 - 16		1,5 - 16		1,5 - 16			
**Sexe**								
Masculin	14	17,5	66	82,5	80	100	0,1130	2,5 [0,9-6,9]
Féminin	6	7,8	71	92,2	77	100		1
**Poids de naissance**								
<2500 grammes	7	36,8	12	63,2	19	100	0,0027	5,6 [1,9-16,7]
=2500 grammes	13	9,4	125	90,6	138	100		1
Moyenne (grammes)	2746,5±566,2		3027,9±505,9		2992,1±520,7		0,0235	
**Notion de réanimation à la naissance**								
Oui	8	53,3	7	46,7	15	100	<0,0001	12,4 [3,8-40,1]
Non	12	8,5	130	91,5	142	100		1
**Administration de la névirapine à la naissance**								
Non	10	66,7	5	33,3	15	100	<0,0001	26,4 [7,6-92,3]
Oui	10	7,0	132	93,0	142	100		1
**Type d'allaitement**								
Artificiel strict	1	6,7	14	93,3	15	100		1
Maternel exclusif	10	8,1	113	91,9	123	100	1,0000	1,2 [0,1-10,4]
Mixte	9	47,4	10	52,6	19	100	0,0204	12,6 [1,3-115,9]

S'agissant des différents types d'alimentation, les enfants nourris strictement au lait artificiel semblent être mieux protégés que ceux nourris exclusivement au lait maternel et ceux nourris par une alimentation mixte, les fréquences de TME ayant été respectivement de 6,7%, 8,1% et 47,4%. Quand on compare la proportion d'enfants infectés nourris strictement au lait artificiel et celle d'enfants infectés nourris exclusivement au lait maternel, aucune différence statistique n'est mise en évidence (p=1,0000); mais quand on la compare à celle d'enfants infectés ayant été nourris de manière mixte, l'analyse statistique donne une différence significative en défaveur de ces derniers (OR=12,6; IC95%: 1,3-115,9; p=0,0204).

## Discussion

### Taux de TME

Le taux global de TME du VIH varie d'un continent à l'autre, d'un pays à l'autre voire même d'une région à l'autre dans un même pays. Notre taux de 12,7% est plus de six fois supérieur à ceux rapportés en 2008 dans les pays développés (moins de 2%) [15, 16] et plus de 12 fois supérieur aux taux actuellement rapportés (moins de 1%) [5]. Notre taux est dans les limites de ceux rapportés dans plusieurs études menées dans les pays africains qui varient de 8 à 23% [17–21]. Mais il faut signaler que des taux faibles parfois aussi proches de celui des pays développés ont été retrouvés dans certains pays d'Afrique sub-saharienne: il était de 2,7% en 2010 et 1,1% en 2011 à Johannesburg (Afrique du Sud) [22], de 4,6% entre 2009 et 2011 à Lilongwe (Malawi) [23], de 4,6% entre 2002 et 2007 à Douala (Cameroun) [24], 3,3% entre 2007 et 2010 à Lusaka (Zambie) [25], 3,28% entre 2006 et 2009 à Abidjan (Côte d'Ivoire) [26] et 1,98% entre 2005 et 2008 à Bamako (Mali) [27]. Le fait que notre fréquence soit plus élevée que celles rapportées dans certains pays à ressources limitées comme le nôtre pourrait être expliqué par plusieurs raisons. Premièrement, les études menées par Mnyani [22], Kim [23] et Chibwesha [25] n'ont inclus que les enfants qui n'avaient que les premiers résultats des tests PCR réalisés à l´âge de 3-12 semaines; leurs résultats ne sont pas directement comparables à ceux d´autres études qui ont suivi les enfants jusqu’à ≥12 mois d’âge car les auteurs reconnaissent que la grande majorité de leur cohorte continuait à être allaitée, et par conséquent, le statut sérologique à VIH final pourrait être modifié. Deuxièmement, les taux de perdus de vue ou des enfants non testés à 12-18 mois d’âge (âge de détermination de leur état sérologique final) dans les études menées par Tsingaing à Douala (Cameroun), Lasme-Guillao à Abidjan (Côte d'Ivoire) et Traoré à Bamako (Mali) variaient autour de 40 à 45% [24, 26, 27]. Ces taux élevés de perdus de vue s'expliqueraient par les facteurs tels que le bas niveau socio-économique des mères, le bas niveau d'instruction et la non-implication du co-géniteur, lesquels facteurs constituent des véritables obstacles au suivi des enfants comme le prouvent plusieurs auteurs africains [22, 28–31]. A ces facteurs, s'ajoute aussi la hantise de certaines mères de découvrir la sérologie positive de leur enfant [26]; d'où la non-réalisation du test de dépistage sérologique à l’âge de ≥12 mois. Mais, malgré toutes ces justifications avancées ci-haut, notre fréquence est plus de 3 à 4 fois supérieur à ceux rapportés dans des études rwandaise [32], camerounaise [33] et sud-africaine [34] ayant suivi des nourrissons nés des mères séropositives au VIH jusqu’à l'arrêt de l'allaitement, traduction probable de l'inefficacité de stratégies PTME dans notre milieu.

### TME et caractéristiques maternelles

Statistiquement dans notre étude, l'analyse univariée montre que la TME n'est pas significativement liée aux caractéristiques sociodémographiques maternelles (l’âge maternel, la parité, l’état-civil, le niveau d’étude et la profession) (p>0,05). Hoffmann trouve que l’âge maternel et la gestité ne constituent pas des facteurs de risque de la transmission verticale [34]. L’étude rwandaise menée par Bucagu note que la primiparité et l’état-civil non marié étaient des facteurs de risque de TME et que par contre l’âge maternel, le niveau d’étude et le niveau de vie n'en étaient pas [35].

### TME et degré d'immunodépression au VIH

L'immunodépression avancée se traduit cliniquement par un stade clinique (selon l'OMS) avancé et la survenue des infections opportunistes, et biologiquement par l'augmentation de la charge virale et de la baisse du taux de CD4. Plus l'immunodépression est avancée, plus le taux de transmission est également élevé. La charge virale plasmatique maternelle élevée reste le principal facteur prédictif biologique à la fois de la TME précoce et tardive [34]. Des essais cliniques ont montré une forte corrélation positive entre la charge virale VIH circulante de la mère pendant la grossesse ou à l´accouchement et le risque de transmission périnatale du VIH, même chez les femmes sous TARV [36]. Cependant, cette variable n'a pas été retrouvée dans notre étude, mais rappelons qu'un faible taux de CD4 est un indicateur de charge virale élevée. Dans notre série, les mères au stade clinique 3 de l'OMS, celles présentant une infection opportuniste et celles ayant un nombre de CD4 <350/mm3 avaient statistiquement un risque élevé de transmettre le VIH chez leurs enfants. L’étude de Ngwende au Zimbabwe a relevé que les enfants nés de mères avec un faible taux de CD4 (<200 cellules/ml) étaient à risque d´infection à VIH [37]. D´autres études ont trouvé des résultats similaires rapportant que les femmes avec un taux de CD4 inférieur à 200 cellules/ml étaient cinq fois plus susceptibles de transmettre le VIH [38–40].

### TME et TARV

Les mères ayant été mises sous TARV tardivement (lors de l'accouchement ou après) ou non mises sous TARV ont transmis plus significativement le VIH chez leurs enfants que celles qui étaient sous TARV bien avant de concevoir. Le temps de mise sous TARV est influencé par la période de dépistage; ainsi nous avons constaté un taux élevé de TME chez les mères ayant été dépisté tardivement (c'est-à-dire à l'accouchement ou pendant l'allaitement). Les résultats de notre étude montrent que le temps de mise sous TARV (ou durée du TARV) constitue un facteur important dans la PTME. Plusieurs études rapportent des faibles taux de TME chez les femmes qui deviennent enceintes sous TARV comparativement aux femmes qui ont débuté le TARV pendant la grossesse [15, 34, 41]. Une étude zambienne montre que, même pour les femmes débutant le TARV pendant la grossesse, la durée du TARV avant accouchement joue un rôle important dans la TME et qu'une durée anténatale de 13 semaines donne un maximum d'efficacité dans la PTME [25].

### TME et paramètres partiaux

Les enfants nés avant la 37ème semaine d'aménorrhée étaient plus infectés que ceux nés à 37 SA ou après (35,7% versus 10,5%). La naissance prématurée constitue ainsi un facteur de risque très important dans la TME. Newell a trouvé un taux de transmission plus élevé si l´accouchement se fait avant 34 semaines [42]. La contamination avant le terme de la grossesse serait facilitée par la faible immunocompétence des foetus, le faible taux d´anticorps acquis transmis et éventuellement, par les infections responsables de l´accouchement prématuré [43]. La rupture prématurée des membranes (RPM) est associée à la transmission verticale du VIH [44]. Nous avons trouvé que 80% des enfants nés après RPM amniotiques étaient infectés contre 8,2% des enfants chez qui les membranes se sont rompues pendant le travail d'accouchement. Une rupture prolongée des membranes amniotiques avant l'accouchement favoriserait la transmission du VIH lors du travail et de l'accouchement [45]. La présence du VIH a été détectée dans les sécrétions génitales: il a été postulé que ces virions pourraient accéder à la cavité utérine durant la grossesse, plus particulièrement durant le travail; la TME périnatale aurait alors lieu par voie ascendante [46]. Le mode d'accouchement n'a pas montré de différence statistiquement significative en ce qui concerne la TME, même observation faite par d'autres auteurs africains [22, 34, 35]. Le manque d´association entre le mode d'accouchement et la TME pourrait s'expliquer par le fait que dans les pays à ressources limitées, les césariennes électives sont peu réalisées et les indications de césarienne en général sont guidées principalement par les nécessités obstétricales plutôt que l´infection à VIH [34]. Plusieurs études ont montré qu'une césarienne élective est un facteur protecteur et entrainerait une réduction significative du risque de TME par rapport à l'accouchement par voie basse [46–48].

### TME et paramètres néonataux

S'agissant du poids de naissance, notre étude rapporte que les enfants infectés avaient un poids moyen inférieur à celui non infectés et que les faibles poids de naissance étaient plus infectés que les enfants normopondérés à la naissance (36,8% versus 9,4%). Contrairement à nos résultats qui sont comparables à ceux des autres auteurs [49–51], Hoffmann ne trouve aucune association significative entre le poids moyen à la naissance, le faible poids de naissance et la TME [34]. Cliniquement, les nourrissons de sexe masculin étaient plus à risque de transmission verticale (17,5% versus 7,6%) bien que ceci n´a pas été statistiquement significatif. Notre constat est similaire à celui de Duri [21] mais contradictoire à celui de Piwoz qui avait observé que la transmission in utero était significativement plus élevé chez les filles que chez les garçons [52]. Les études de Thorne et de Tonwe-Gold notent aussi que les nourrissons de sexe féminin comportent un risque plus élevé de TME que leurs homologues masculins [49, 53]. Si cette différence est due à la susceptibilité de sexe ou au hasard, elle reste cependant discutable et mérite d’être approfondie. Dans cette étude, le risque de TME était de 66% chez les enfants n'ayant pas reçu de prophylaxie contre 7% pour les nourrissons qui ont reçu la névirapine à la naissance. Nos résultats sont comparables à ceux rapportés par d'autres études antérieures faisant état d´un bénéfice plus élevé de la prophylaxie antirétrovirale pour les nourrissons exposés [34, 54–56]. Le diagnostic précoce du VIH chez les nourrissons offre une chance unique de renforcer le suivi des enfants exposés au VIH et initier un traitement précoce pour ceux qui sont infectés par le VIH. En cas de non traitement, l´infection à VIH chez les enfants est associée à des taux de mortalité très élevés [18, 57].

### TME et allaitement

Les lignes directrices de PTME en République Démocratique du Congo approuvent les directives de l´OMS sur l´alimentation du nourrisson. Ainsi, la question de l'allaitement dans le contexte africain reste un dilemme pour beaucoup de femmes non-allaitantes, au risque d’être rejetées ou stigmatisées comme le montre Oladokun dans son étude [58]. Ceci aboutit finalement à des pratiques d´alimentation du nourrisson inappropriées comme en témoigne le taux de l´alimentation mixte pratiqué par les mères séropositives dans cette étude. Beaucoup de femmes, après avoir choisi l'allaitement artificiel, pratiquent l'allaitement mixte qui est la méthode la plus risquée dans la transmission verticale. Nous avons constaté que l´infection à VIH était significativement associée à une alimentation mixte (p < 0,05). Ce constat est similaire à ceux rapportés dans plusieurs études [20, 38, 57, 58]. Il est prouvé que l´alimentation mixte, par rapport à l´allaitement maternel exclusif et l´alimentation artificielle stricte, est associée à un risque accru de transmission du VIH [20, 59]. Au cours de l´alimentation mixte, les facteurs immunitaires bénéfiques du lait maternel sont probablement contrebalancés par les dégâts à la paroi intestinale de l´enfant par des contaminants ou des allergènes dans les aliments mixtes [60]. Par contre, l´allaitement maternel exclusif favorise le maintien de l´intégrité de barrière gastro-intestinale de l´enfant (considérée comme le principal mode d´infection). Récemment, il est établi que les facteurs immunologiques dans le lait maternel sont susceptibles de réduire l´activité virale dans le lait humain [61].

**Limites de l’étude**: il faudra souligner que la mise en œuvre de ce schéma thérapeutique butait sur des difficultés pratiques surtout en ce qui concerne certaines mères qui d'une part, pour les non éligibles ayant passé moins d'une semaine à la maternité, ne poursuivaient pas leur prophylaxie ARV après leur sortie de la maternité, et d'autre part, ne continuaient pas à administrer la NVP chez leurs enfants après leur sortie de la maternité pour des raisons diverses.

## Conclusion

De manière globale, notre étude montre que la stratégie de l'option A dans la PTME qui est de mise actuellement dans notre pays n'offre pas de de grand garanti quant au risque de la TME. Ce risque reste encore très élevé soit 12,7% alors que l'OMS prône « Zéro Nouvelles Infections », surtout chez les enfants dès l'année 2015. Il est donc important de penser à renforcer les occasions de « tracking » de toutes les femmes enceintes afin de leur offrir un paquet complet de prise en charge du VIH lorsqu'il est établi qu'elles sont VIH positives. A l'instar d'autres pays, le passage à l'opéralisation de l'option B+ comme nouvelle stratégie dans la PTME s'avère plus qu'indispensable, car elle permet de mettre directement une femme sous TARV dès l'instant où elle est dépistée VIH+ sans tenir compte de sa classification biologique.
